# Clustered Regularly Interspaced Short Palindromic Repeats (CRISPR)-Cas genome editing transforming crop stress tolerance for global food security

**DOI:** 10.7717/peerj.21450

**Published:** 2026-07-01

**Authors:** Wen XiuJuan, Muhammad Faisal, Sher Muhammad, Ali Aslam, Muhammad Khuram Razzaq, Ashir Masroor, Sun Yefang, Afnan S. Quronfulah, Muna Abdul-Rahman Al-Malki, Hanan El Sayed Osman

**Affiliations:** 1 Academy of Agricultural Science, Shaoxing, China; 2 Food Safety & Consumer Protection (FS&CP) Department, Government of Punjab, Pakistan; 3 Faculty of Agriculture and Veterinary Sciences, Superior University, Lahore, Pakistan; 4 Sub-Campus Burewala-Vehari, University of Agriculture, Faisalabad, Pakistan; 5 Department of Biology, College of Science, Umm Al-Qura University, Makkah, Saudi Arabia; 6 Botany and Microbiology Department, Faculty of Science, Al-Azhar University, Cairo, Egypt

**Keywords:** CRISPR-Cas, Genome editing, Climate-resilient crops, Food security, Biotic and abiotic stress tolerance, Sustainable agriculture

## Abstract

Climate change increasingly threatens global crop productivity by intensifying drought, salinity, temperature extremes, and biotic stresses. Developing climate-resilient cultivars has therefore become a central objective in modern crop breeding programs. Conventional breeding approaches are often limited by complex trait inheritance and long selection cycles, particularly for polygenic stress-adaptive traits. Clustered Regularly Interspaced Short Palindromic Repeats (CRISPR)-associated protein (Cas) genome editing genome editing provides a precise and efficient platform for targeted manipulation of genes controlling stress tolerance, yield stability, and adaptive performance. This review synthesizes recent advances in CRISPR mediated improvement of resilience to major abiotic stresses (drought, salinity, heat, and cold) and biotic stresses (fungi, bacteria, viruses, and insects) across important cereal, legume, and horticultural crops. Emphasis is placed on the editing of transcription factors, signaling regulators, susceptibility genes, and redox-associated pathways that enhance physiological and molecular stress adaptation. Furthermore, the integration of CRISPR with genomics, transcriptomics, proteomics, metabolomics, genome-wide association studies, high-throughput phenotyping, and artificial intelligence-driven prediction tools is accelerating precision breeding strategies. Despite remaining challenges related to off-target effects, delivery systems, and regulatory frameworks, genome editing represents a transformative approach for advancing climate-resilient crop development and sustainable agricultural production.

## Introduction

Since 1970, the Food and Agriculture Organization (FAO) of the United Nations (UN) has revised its definition of food security several times, reflecting shifting global priorities. It was initially defined in 1974 as a situation in which every person constantly has economic and physical access to enough food that fulfills their preferences and dietary needs. This concept was broadened in 1983 to emphasize the consistent availability of sufficient nutritious food to lead a healthy life. According to Antle (2011), food is essential for survival and health, and food security involves more than just food availability, it is linked to human health and development. It is crucial to ensure the availability of safe and nutritious food for the world’s rapidly growing population (Li & Song, 2022). Food security is linked to the complex interrelation between environmental factors and human activities in every step of the food chain, from production and distribution to consumption (Rahman et al., 2025). Malnutrition in all its forms, including hunger, constitutes one of the most serious challenges; about 9.1% of the global population suffered from hunger in 2023 (Gomes et al., 2026). Within this context, various challenges can be addressed through sustainable agricultural approaches such as conservation agriculture, climate-smart agriculture, and agroecology, which offer effective contributions to increasing productivity while preserving ecological balance and resilience (Olarewaju et al., 2025). However, effectively addressing food security will require an integrated multi-dimensional approach that incorporates policy reforms, investment in climate-smart technologies, research and innovation, and infrastructure development, among others (Simane et al., 2025). In 2015, the United Nations (UN) developed the SDGs as part of the UN 2030 Sustainable Development Agenda, comprising 17 universal goals aimed at ending poverty, protecting the environment, and improving human life. Specifically, Goal 2 commits to zero hunger, access to nutritious food, and sustainable agriculture (Vågsholm, Arzoomand & Boqvist, 2020; Rahman et al., 2025).

Climate change has appeared as a critical threat to world food security (Hasegawa et al., 2018). Climate change occurs due to climate variability as well as changes in the environment that affect the agricultural sector, and the cause for the disruptions in the sector arises because of climate variability impacting agricultural productivity (Anshida et al., 2026). The impacts of climate change, combined with rapid population growth, acutely increase demand for resources and place widespread stress on global food systems. Such changes negatively affect the agroecosystem, leading to the emergence of diverse biotic and abiotic stresses that damage plant physiology and metabolism (Mirzabaev et al., 2023). Major biotic stresses, such as pests, are critical, while abiotic stresses, including temperature extremes, salinity, heavy metal toxicity, and drought, also cause serious yield losses (Jamil et al., 2024; Alwutayd et al., 2024; Tao & Chen, 2025). In response to these challenges, the development of climate-resilient crops and varieties bred or engineered for tolerance to the prevalent associated stresses of drought, heat, flooding, and pest infestation is a high priority on the global agricultural agenda. Those crops indicate one of the cornerstones of sustainable food security. The successful development of these crops depends on integrating molecular breeding approaches with high-throughput phenotyping and multidisciplinary scientific approaches (Rivero et al., 2022; Kopeć, 2024). Molecular breeding utilizes sophisticated methods such as marker-assisted selection, genomic selection, and genome editing to enhance crop yield and quality. The process entails the application of cutting-edge technologies like CRISPR, RNA interference, and multi-omics for precise trait modification and selection. This process ensures improved crop productivity through sustainable crop development practices (Khan et al., 2024).

Traditional breeding approaches are both time-consuming and labor-intensive, as the complexity of the plant genome severely limits the number of new varieties that can be developed to exhibit desirable traits (Krishna et al., 2023). This changed significantly with the recent discovery of modern genome-editing technologies, which have equipped breeders with tools that enable the modification of plant DNA in a precise, efficient, and rapid manner. Clustered Regulatory Interspaced Short Palindromic Repeats (CRISPR) appeared as the most versatile and adopted approach for genome manipulation across species (Prabhu et al., 2023). The CRISPR system enables the production of genetically edited plants without introducing transgenes (Wada et al., 2020). In addition, compared to zinc-finger nuclease (ZFN) and Transcription Activator-Like Effector Nuclease Technology (TALENs), CRISPR-based tools are easier and more efficient to design, much more cost-effective, and therefore an indispensable part of modern crop improvement programs (Chen et al., 2024). CRISPR advanced editing systems, such as prime editors and base editors, improve precision by enabling single-nucleotide modifications or small insertions/deletions without inducing double-stranded DNA breaks. Such innovations are crucial to address emerging challenges in food security, climate change, and adaptation to environmental stresses. Moreover, editing genes *HsfA1*, *ERECTA*, and gene families/pathways such as SOS, HSP, NHX, DREB, and SOS conferred significant tolerance to salinity, heat, and drought stresses in crop plants (Chavhan et al., 2025).

Moreover, genome engineering improves key physiological traits, including heat shock response, membrane stability, ion homeostasis, water-use efficiency, photosynthetic performance, osmotic adjustment, and oxidative stress tolerance (Avendaño et al., 2025). Gene editing, combined with phenomics, genomics, and machine learning (ML)/artificial intelligence (AI) platforms, could accelerate next-generation breeding. However, certain challenges, such as off-target mutations, regulatory barriers, and delivery inefficiencies, must be systematically addressed for the safe and widespread dissemination of such technologies. Hence, CRISPR-Cas based and prime editing technologies advance precise, powerful strategies to enhance crop resilience and support sustainable agriculture (Razzaq et al., 2023; Mansoor et al., 2025; Chavhan et al., 2025). This review highlights how CRISPR genome-editing technology can transform global food security by developing crop varieties that can tolerate the stresses of a changing climate. This review explains how precision genome editing enhances crop tolerance to biotic and abiotic stresses associated with climate change. Further, this review covers recent developments, applications, and the adoption of CRISPR in combination with breeding and multi-omics. Additionally, it discusses technical challenges, regulatory issues, and future perspectives for achieving agricultural sustainability.

### Target audience

This review is intended for plant biotechnologists, molecular geneticists, crop breeders, plant physiologists, and agricultural researchers working on stress biology and crop improvement. By synthesizing recent advances in CRISPR-Cas genome editing and systems biology approaches, the article provides a comprehensive resource for researchers seeking both mechanistic insights and practical strategies for developing climate-resilient crops and improving agricultural sustainability under changing environmental conditions.

### Survey/search methodology

To ensure comprehensive and balanced coverage, a systematic literature search was conducted using major scientific databases including Scopus, Web of Science, PubMed, and Google Scholar. Priority was given to peer-reviewed research articles, reviews, and authoritative reports focusing on genome editing, stress biology, and crop resilience. The research included recent advances in CRISPR-Cas technologies as well as integrative omics and artificial intelligence-assisted breeding approaches relevant to crop stress adaptation.

### Inclusion and exclusion criteria

Clearly defined inclusion and exclusion criteria were applied to enhance transparency and reduce bias.

### Included studies

• Research on CRISPR-Cas applications in crop improvement
• Studies addressing abiotic stress tolerance (drought, salinity, heat, cold)
• Studies addressing biotic stress resistance (fungi, bacteria, viruses, insects)
• Multi-omics integration (genomics, transcriptomics, proteomics, metabolomics)
• Artificial intelligence (AI) assisted breeding and systems biology approaches

### Excluded studies

• Non-plant genome editing applications
• Studies not addressing stress resilience
• Articles lacking mechanism or applied relevance to crop improvement

The iterative screening process followed systematic review principles to ensure rigor and minimize publication bias.

### Search strategy and keywords

Primary and secondary keywords were combined using Boolean operators to maximize retrieval of relevant studies. General search engines (Google, Bing, DuckDuckGo) were also used to identify recent preprints and emerging literature not yet indexed in major databases.

### Primary key terms included

CRISPR-Cas AND crop stress tolerance

Genome editing AND climate-resilient crops

CRISPR AND drought tolerance in crops

CRISPR AND salinity stress

CRISPR AND heat stress

CRISPR AND disease resistance in crops

CRISPR AND transcription factors in stress response

Base editing OR prime editing AND crop improvement

Multi-omics AND genome editing

AI-driven breeding AND CRISPR

CRISPR AND sustainable agriculture

Genome editing AND food security

## Plant Physiological Responses to Stresses

Plant exposure to stresses is known to affect their physiology, and crop yield potential. The plant response to stress is a complex multilevel one including sensing, signaling, transcription, transcript modification, protein synthesis, and protein modification (Zhang et al., 2022). Stress factors like biotic and abiotic, drought, salinity, and temperature extremes have negative impacts on plant physiology, metabolism, and growth processes due to osmotic stress, toxic ions, and oxidative stress. Plants have evolved to cope with stress using various physiological mechanisms such as osmoregulation, stomatal control, antioxidants, and signaling systems such as calcium, reactive oxygen species, and abscisic acid (Zhang et al., 2023; AL-Huqail et al., 2023). The movements of stomatal guard cells are an integral part of the plant defense mechanism. Although the stomata open to facilitate the exchange of gases and water, they are one of the main avenues that allow bacteria entry into plants. The defense mechanism that plants use against this invasion includes activating PAMP-triggered stomatal closure in order to prevent entry of the pathogens. In turn, the pathogens developed ways to suppress this defense mechanism through disruption of SA signaling, MPK3 pathway, OST1 kinase, ABA signaling, proteolysis, proton pumps, K^+^ uptake, and starch metabolism in guard cells (Balasubramaniam et al., 2023). Plants adapt to stress by physiological changes, such as maintaining osmoregulation by accumulating solutes like proline and sugars to ensure proper hydration levels and turgidity in cells. They control their ion balance and compartmentalization, as well as stomatal closure, to minimize the loss of water when exposed to stress. Their antioxidant defense mechanisms help eliminate ROS and prevent oxidative damage to their cellular components. Moreover, stress signal transduction pathways, which involve ABA, Ca^2^^+^, and protein kinases, regulate genetic and biochemical changes that improve survival (Hina et al., 2024). Heat, high light, or wounding perception is a major trigger for systemic signals that include electrical, calcium, and ROS, among others, which spread to systemic tissues and cause systemic acquired acclimation (SAA) or systemic wound response (SWR). These waves travel within the plant through the vascular bundles and mesophyll cells to enable systemic signaling (Zandalinas & Mittler, 2021). Furthermore, plants modify their physiology, adjust their roots’ growth and architecture, and shut down stomata in the upper parts of their bodies in response to the soil’s moisture stress. This tissue-based response alters the flow of cellular information, leading to early flowering or growth retardation, among others, and usually low productivity. Studies of physiology and molecular biology have revealed that phytohormones play a vital role in controlling the response to water deficit (Gupta, Rico-Medina & Caño Delgado, 2020). Abscisic Acid (ABA) is a hormone that controls plants’ reactions to dehydration and maximizes the efficiency of water use by plants. Dehydration stimulates the production of ABA in particular tissues of the plant body, yet, the process of production is faster in leaves than roots (McAdam & Brodribb, 2018). The excess accumulation of ABA leads to the activation of signaling cascades. In response to stress conditions, ABA performs its role by conducting signal crosstalk among other pathways (Kalladan et al., 2017; Kuromori, Seo & Shinozaki, 2018). The sensitivity of the ABA receptor PYR1 was improved through engineering to increase drought tolerance in Arabidopsis and tomatoes using the agrochemical mandipropamid (Park et al., 2015). The virtual screen for ABA receptors’ agonists resulted in the discovery of a bioactive ABA analog known as opabactin. This molecule facilitates the process of activating ABA receptors’ downstream signaling pathways by promoting SnRK2 activation (Cai et al., 2014). Yet another possibility is an AP2/ERF transcription factor called TINY, which counteracts the growth-inducing effect of brassinosteroids. While BES1 promotes growth by inhibiting TINY, BIN2 targets TINY *via* phosphorylation to stabilize it and induce stomatal closure under ABA conditions (Xie et al., 2019). Hence, under stress, plants shift resources from growth to survival, processes like osmolyte accumulation, stomatal closure, and ABA signaling, conserve water and protect cells, but they also reduce photosynthesis, cell division, and energy availability, leading to lower growth and productivity.

## CRISPR Compared with Other Genome-Editing Technologies

Wheat productivity and quality is important for world food security, as it is a staple crop providing essential nutrients and calories worldwide. CRISPR provides a fast and precise approach to develop high yielding, climate-resilient crops essential for meeting global food demands by 2050 (Ahmed et al., 2021; Elsharawy & Refat, 2023). Genome editing refers to a set of advanced molecular techniques that allow researchers to make precise, targeted changes to an organism’s DNA, thereby allowing the insertion, deletion, or alteration of genes at specific sites in the genome (Ahmad, 2023). The combination of CRISPR with multi-omics approaches and smart breeding technologies for accelerating climate-resilient crop development is summarized in Table 1. Over time, several platforms for genome engineering have been developed, including ZFNs and TALENs. Recently, however, the CRISPR system has completely changed the landscape of this field. Due to its speed, affordability, ease of design, and high accuracy, CRISPR quickly replaced previous technologies and became extremely popular among scientists in a very short time (Gupta et al., 2019).

**Table 1:** Integration of CRISPR with omics and smart breeding tools.

**Approach**	**Function in CRISPR research**	**Benefits for climate-resilient crop development**	**Example/application**	**References**
Genomics & Transcriptomics	Identify target genes and regulatory networks	Precision identification of stress-responsive loci	*DREB, HSP, NHX* genes	Chavhan et al. (2025)
Proteomics	Characterize stress-related proteins	Uncover molecular pathways altered by editing	Heat shock protein profiling	Singh et al. (2024)
Metabolomics	Quantify metabolites under stress	Identify biomarkers of stress tolerance	Osmoprotectant accumulation	Razzaq et al. (2023)
Phenomics	Link genotype to phenotype	Real-time monitoring of edited plant performance	High-throughput field phenotyping	Arya et al. (2022); Kaya (2025)
AI/ML Integration	Predict off-targets, optimize gRNA design	Enhances accuracy and editing success	AI-guided CRISPR design platforms	Zhang & Wang (2023); Eftekhari, Ma & Orlov (2024)
GWAS + CRISPR	Validate QTL-linked genes	Accelerates breeding for complex traits	For example, validation of GWAS loci for drought tolerance	Razzaq et al. (2023)

### Zinc Finger Nuclease

Targeted genetic modification across many important model organisms was once a challenging task. During the past few years, this has changed dramatically with the advent of ZFN technology. Genome-editing tools, including but not limited to ZFNs and TALENs, with FokI restriction endonuclease catalytic domains that create DSBs, have played a significant role in advancing genome engineering. ZFNs are artificial DNA-binding proteins consisting of two domains linked by a linker sequence. The first domain is a zinc finger DNA-binding domain that recognizes approximately 24 base pairs of a specific DNA sequence, and the second domain, derived from FokI, functions as a DNA-cleaving component that cuts a spacer region of approximately 5–7 base pairs. Each zinc finger binds to three base pairs of DNA. Each zinc finger is composed of approximately 30 amino acids coordinated by a single zinc atom. The cleaving function of this domain is not specific (Kim et al., 1997; Urnov et al., 2010).

### Transcription Activator-Like Effector Nucleases

TALENs are constructed similarly to ZFNs. Like ZFNs, they comprise two primary domains: an N-terminal transcription activator-like effector (TALE) DNA-binding domain and a C-terminal FokI restriction endonuclease domain. A linker sequence connects these two domains, while a spacer region of approximately 12–25 base pairs (bp) separates the two binding sites. TALENs induce double-strand breaks (DSBs) at specific target sites when TALENs bind to their respective DNA sequences, located on opposite strands. TALENs’ DNA-binding region contains tandem repeat domains that recognize and bind to a specific nucleotide. Typically, a pair of TALENs targets a DNA sequence ranging from 12 to 25 bp. Limitations in the complex design, technical difficulties, and high costs associated with both TALEN and ZFN based genome engineering systems have constrained their application despite substantial advancements in functional genomics. Therefore, there was a pressing need to develop another genome-editing system that is simpler, more accurate, and less costly. The CRISPR system was thus developed as a formidable and efficient alternative for precise modification of genomes (Joung & Sander, 2013; Gupta et al., 2019).

### CRISPR technology

Originating from a prokaryotic RNA-guided defense system, the CRISPR technology has rapidly become a leading platform for precise genetic modification (Jain, 2015). The CRISPR system’s simplicity, engineering, and amenability to multiplexing make it the system of choice compared to ZFNs and TALENs. These benefits enable more efficient targeted genome editing for diverse biological research (Eid & Mahfouz, 2016). Several studies and reviews have elucidated on the innovation, usefulness and mechanism of the CRISPR system in various organisms (Kumar & Jain, 2015; Paul & Montoya, 2020; Marino et al., 2020). CRISPR, as a genome-editing system, enables researchers to explore and modify functional regions of the plant genome. This approach has been applied to modify both regulatory genes and cis-acting elements to enhance plant tolerance to various abiotic stresses, including drought, heat, and salinity (Ajithkumar et al., 2025). CRISPR consists of a two-component structure that mediates gene editing: one component is the single guide RNA (sgRNA), while the second is the Cas9 nuclease (Ansori et al., 2023; Razzaq et al., 2024). Guided by the sgRNA, Cas9 locates the targeted site in the genome and introduces a double-stranded break (DSB) in the DNA. The synthetic RNA, including the crRNA and tracrRNA, ensures the accuracy of target recognition. At the 5′ end of the sgRNA, the first 20 nucleotides that specifically bind the target DNA sequence are located next to a PAM, which is usually a short, specific sequence. The PAM sequence is a small, conserved region of just a few base pairs that immediately follows the genomic target the system intends to cut. The Cas9 enzyme, most commonly used as the SpCas9 variant from Streptococcus pyogenes, is a multidomain nuclease comprising approximately 1,368 amino acids. SpCas9 induces a blunt-ended double-strand break guided by the sgRNA, after which the DNA is repaired *via* diverse host cell repair systems, primarily homology-directed repair or nonhomologous end joining (Ajithkumar et al., 2025).

Cas proteins that have been discovered, the following are the most commonly used for genome editing in plants:

• Cas9 (type II): Among the various CRISPR-associated nucleases, the Cas9 nuclease remains the most widely used for genome editing applications. The SpCas9 enzyme recognizes the NGG protospacer adjacent motif and cleaves the DNA at the intended locus, generating blunt double-strand breaks. To enhance the precision of genome editing and reduce unintended modifications, multiple high-precision Cas9 variants, including eSpCas9 and SpCas9-HF1, have been engineered to reduce off-target cleavage significantly (Gürel et al., 2020).
• Cas12a (Cpf1, type V): Cas12a, previously referred to as Cpf1, is classified as a Type V CRISPR enzyme that acts as an alternative to Cas9 for genome editing. Unlike SpCas9, Cas12a recognizes thymine-rich protospacer adjacent motifs (PAMs), typically TTTV (where V can be A, C, or G). Cas12a creates staggered double-strand breaks (DSBs) with sticky ends other than blunt cuts, which confer advantages for targeted gene insertions. This requires a simpler structure of guide RNA, and, due to its small size and high efficiency in editing, Cas12a is also very suitable for plant genome transformation and multiplex editing applications (Swarts & Jinek, 2019).
• Cas13 (type VI): Although other Cas proteins target DNA, Cas13 has the distinctive feature of targeting and cleaving RNA molecules specifically, thus allowing post-transcriptional gene silencing with high precision. Cas13 is therefore widely used to silence RNA viruses, regulate gene activity, and modulate transcripts involved in stress responses across various organisms, including plants (Hillary & Ceasar, 2023).
• Cas 14 and CasΦ: CRISPR-associated nucleases that are ultra compact, recognized by their small size, and particular functional properties. Cas 14 can detect single-stranded DNA (ssDNA) and cleave. Cas Φ possesses an extremely compact structure and multifunctionality. Their size and efficiency make both nucleases particularly helpful for applications, including plant transformation vectors (Ullah et al., 2023).

## Developing Climate-Resilient Crops

**Figure 1:**
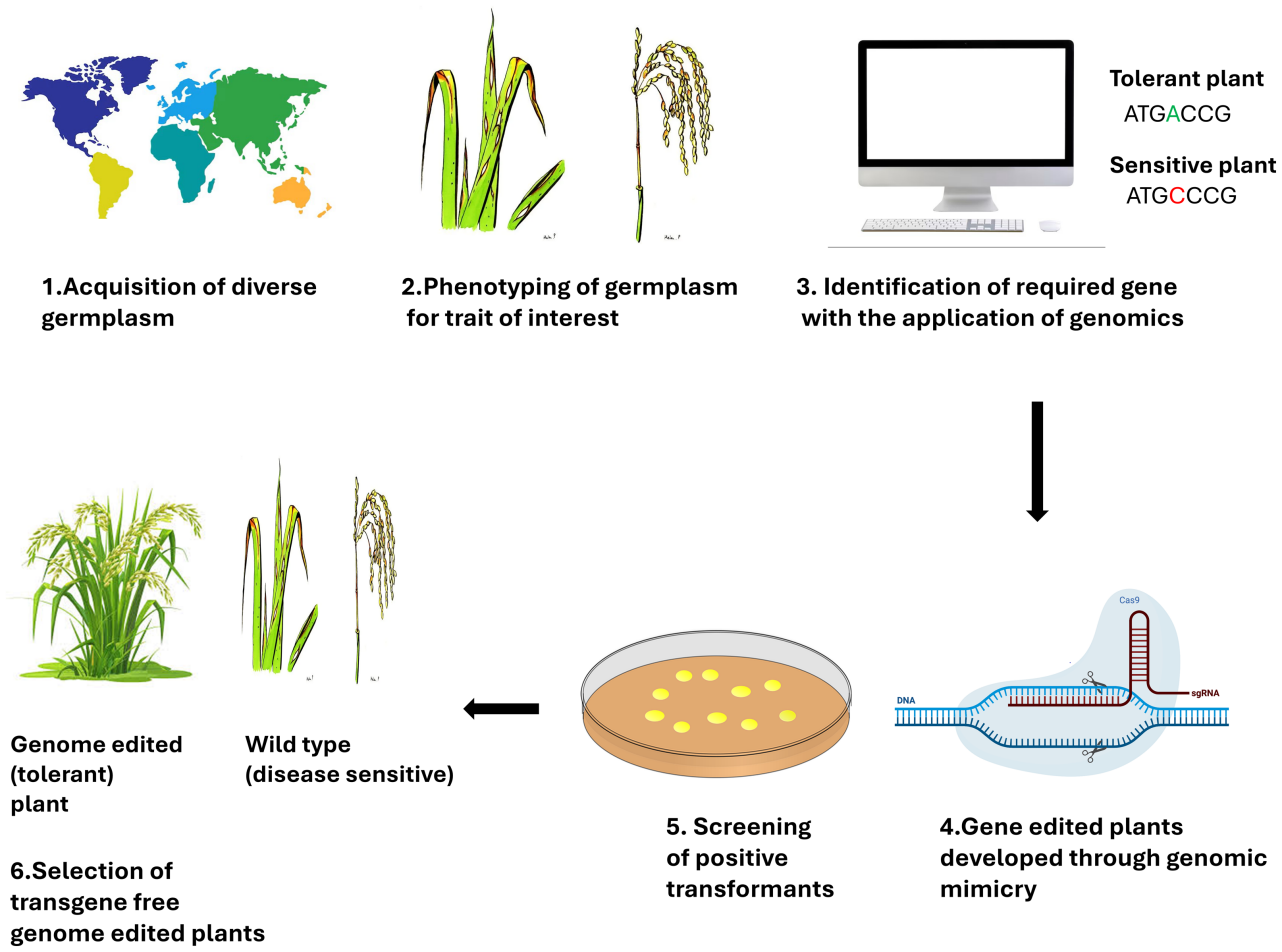
Schematic representation of the stepwise application of CRISPR genome editing to develop climate-resilient crops for food security. (1) Collection of genetically diverse crop accessions and wild relatives to ensure broad genetic variability. (2) Evaluation of germplasm under specific stress or environmental conditions to identify tolerant and susceptible genotypes for the trait of interest. (3) Integration of genomics to pinpoint genes associated with the trait of interest. (4) Targeted knockout or modification of target genes to enhance tolerance and improve desired traits. (5) Molecular and phenotypic screening of regenerated plants to confirm successful genome edits. (6) Selection of transgene free genome edited climate resilient crop plants. Figure created using BioRender.com and Powerpoint.

Climate change increasingly disrupts global food production systems (Abbas et al., 2023). Droughts, floods, heatwaves, and cold snaps may severely threaten crop yields in most regions of the world, while the persistent rise in atmospheric carbon dioxide levels further complicates agricultural stability (Kopeć, 2024; El-Mahdy et al., 2024). Among these challenges, the United Nations is leading the way with a new approach called climate-smart agriculture, which aims to achieve food security through sustainable farming that also enables adaptation. At the core of this strategy is the creation of crops that are resilient to climate stress, scientifically defined as plant varieties or cultivars resistant to environmental stress. Modern agriculture needs diversification of crop species and cultivars to enhance resilience. Accomplishing this requires an integrated use of advanced molecular breeding techniques and field phenotyping technologies to detect and select superior genotypes accurately. In addition, effective progress needs close collaboration among plant breeders and researchers from various scientific disciplines to stimulate innovation in sustainable crop improvement (Kopeć, 2024). Figure 1 illustrates that climate-resilient varieties will require exploration of genetic diversity, genomics, target editing, and trait improvement.

### CRISPR mediated improvement of biotic stress tolerance in crop plants

CRISPR serves not only as a powerful tool for functional genomics research but also as a promising approach for the genetic enhancement of crop varieties (Kafle, 2023). Biotic stresses from bacteria, fungi, and viruses account for roughly 20–40% of crop yield losses worldwide (Li & Song, 2022). Enhancing plant resistance to pathogens is an effective approach to mitigate disease impacts and boost overall crop yields (Hina et al., 2024). The CRISPR genome editing system has been successfully applied to several cereal crops, including rice, maize, and wheat (Ndudzo et al., 2024). To date, most genome editing work has focused on biotic stresses, particularly plant diseases. Recent studies by Malik, Rejab & Aziz (2026) demonstrated that the combination of multile resistant genes (*Pi2*, *Pi9*, *Piz*) using marker-assisted selection is effective for developing rice varieties with broad-spectrum and durable resistance to blast disease. Moreover, the CRISPR-Cas9 system was applied to create rice yellow mottle virus (RYMV) resistance by editing the rice genome. Editing the *OsCPR5.1* (rymV2) gene led to significant resistance (Arra et al., 2024). CRISPR-Cas9 system was applied to explore and edit brown plant hopper (BPH) resistance in rice through *OsMYB22* gene editing. MYB22KO and MYB22OE lines indicated the importance of *OsMYB22*, which is controlled by *OsmiR319b*, in BPH resistance (Sun et al., 2024).

For instance, in wheat, knocking out all three *EDR1* homologs simultaneously using the CRISPR genome-editing technique resulted in Taedr1 mutant lines that showed enhanced resistance to powdery mildew (Zhang et al., 2017). Similarly, pests are estimated to cause 20–40% of global crop yield losses (Douglas, 2018), not only by damaging plants directly but also by transmitting a wide range of plant pathogens (Razzaq et al., 2023). In the meantime, however, insect-induced losses have substantially increased due to global climate change (Deutsch et al., 2018), though overreliance on pesticides still carries serious environmental risks (Subedi, Poudel & Aryal, 2023). Disruption of *OsCYP71A1*, a gene encoding serotonin biosynthesis, enhanced the accumulation of salicylic acid in rice and provided increased resistance against stem borers and plant hoppers (Lu et al., 2018). Also, CRISPR mediated knockout of *GMCDPK38* yielded mutant lines resistant to the common cutworm (Li et al., 2022).

CRISPR mediated mutations in *OsNRAMP1* rice lines resulted in the increase of H_2_O_2_ and the enhancement of SOD activity, while CAT activity was diminished. Under these conditions, the plants exhibited tolerance to a wide range of bacterial and fungal pathogens (Chu et al., 2022). In another study, CRISPR induced modifications in the tomato susceptibility gene *SlDMR6-*1 demonstrated resistance to numerous pathogens, fungi, bacteria, and oomycetes (Thomazella et al., 2021). Bacterial diseases in plants remain difficult to control due to the challenge of early diagnosis, and only a few chemical treatments are available. Nevertheless, by employing CRISPR for genome modification, various crops have shown enhanced resistance against bacterial infection. An example is *OsSWEET13*, an *S* gene in plants that encodes a sucrose transporter and plays a key role in interactions with pathogens. The effector protein PthXo2 from *Xanthomonas oryzae* can activate *OsSWEET13* expression in host tissues, thereby triggering disease susceptibility. Modification of the *OsSWEET13* promoter *via* CRISPR-Cas9 resulted in rice plants resistant to bacterial blight (Zhou et al., 2015). Another example is that *Pseudomonas syringae*, a bacterium that causes bacterial leaf spot disease, promotes infection by inducing stomatal opening. The JAZ protein is a coreceptor for coronatine; thus, CRISPR-Cas9 editing of *SlJAZ2*, removing its JAZ domain, in tomato plants provided effective resistance against bacterial leaf spot (Ortigosa et al., 2019).

Rice blast ranks among the most damaging fungal diseases of rice worldwide. Increased resistance to this disease was achieved by disrupting the *OsSEC3A* gene in rice using CRISPR (Ma et al., 2018). Powdery mildew is another disease that severely limits crop yield. By using CRISPR-Cas9, scientists successfully disrupted all three *TaMLO* alleles in wheat, resulting in improved resistance to powdery mildew (Wang et al., 2014). A mutation in the susceptibility gene *EDR1* enhanced *Arabidopsis* tolerance to powdery mildew infection (Frye, Tang & Innes, 2001). Table 2 summarizes CRISPR-based genome editing for developing crop resistance against biotic stresses.

**Table 2:** Applications of CRISPR-mediated genome editing for improving biotic stress resistance in crops to strengthen food security.

**Crop**	**Target gene(s)**	**Editing approach**	**Pathogen type**	**Key outcome**	**Reference**
Wheat	*TaEDR1*	CRISPR-Cas9 knockout	Fungal (Powdery mildew)	Enhanced powdery mildew resistance	Zhang et al. (2017)
Rice	*OsCYP71A1*	CRISPR-Cas9 knockout	Insect (Hopper, Borer)	Increased salicylic acid and insect resistance	Lu et al. (2018)
Soybean	*GmCDPK38*	CRISPR-Cas9 knockout	Insect (Cutworm)	Improved resistance to common cutworm	Li & Song (2022)
Rice	*OsSWEET13*	CRISPR-Cas9 promoter knockdown	Bacterial blight (*X. oryzae*)	Resistance to bacterial blight	Zhou et al. (2015)
Tomato	*SlJAZ2*	CRISPR-Cas9 editing	Bacterial leaf spot (*P. syringae*)	Resistance to bacterial infection	Ortigosa et al. (2019)
Tomato	*SlDMR6-1*	CRISPR-Cas9 knockout	Bacterial, fungal, oomycete	Broad-spectrum disease resistance	Thomazella et al. (2021)
Rice	*OsNRAMP1*	CRISPR-Cas9 knockout	Bacterial & Fungal	Increased ROS and SOD activity, enhanced resistance	Chu et al. (2022)
Rice	*OsSEC3A*	CRISPR-Cas9 knockout	Fungal (Rice blast)	Improved resistance to rice blast disease	Ma et al. (2018)
Wheat	*TaMLO*	CRISPR-Cas9 knockout	Fungal (Powdery mildew)	Powdery mildew-resistant wheat plants	Wang et al. (2014)
Tomato	*miR482b, miR482c*	CRISPR-Cas9 knockout	Fungal (Late blight)	Double mutants showed higher resistance	Hong et al. (2021)
Citrus	*CsLOB1*	CRISPR-Cas9 modification	Bacterial (Citrus canker)	Enhanced resistance to canker in sweet orange	Peng et al. (2017)
Benth	Viral genome	CRISPR-Cas9 targeting	Begomovirus	Resistance through genome cleavage of the virus	Ali et al. (2015)
Arabidopsis, Tobacco	RNA virus genomes	FnCas9-sgRNA targeting	RNA viruses (CMV, TMV)	Molecular immunity against RNA viruses	Zhang et al. (2018)
Cotton, Tomato	CLCuKoV, TYLCV	CRISPR-Cas9 targeting	DNA viruses	Resistance to multiple viral infections	Zaidi et al. (2016)

Tomato late blight, by *Phytophthora infestans*, ranks among the most severe fungal diseases affecting tomatoes. Small RNAs, including miRNAs, are crucial for controlling plant immune responses by suppressing specific target genes. Simultaneous knockout of *miR482b* and *miR482c* using a multiplex CRISPR editing approach generated double mutants that were more resistant than single mutants, revealing a new pathway through which miRNAs influence resistance to fungal pathogens (Hong et al., 2021). Similarly, Peng et al. (2017) showed that CRISPR-Cas9 editing of the *CsLOB1* promoter region, namely EBEPthA4, in the Wan Jincheng orange (*Citrus sinensis* Osbeck) significantly improved citrus canker resistance. The CRISPR-Cas9 has additionally been applied to improve plant resistance to begomoviruses. In these experiments, CRISPR targeted the host cell nuclei, where it cleaved the viral genome during replication. The engineered sgRNA molecules were introduced into *Nicotiana benthamiana* plants engineered to overexpress Cas9 through a tobacco rattle virus (TRV) based delivery system (Ali et al., 2015).

**Figure 2:**
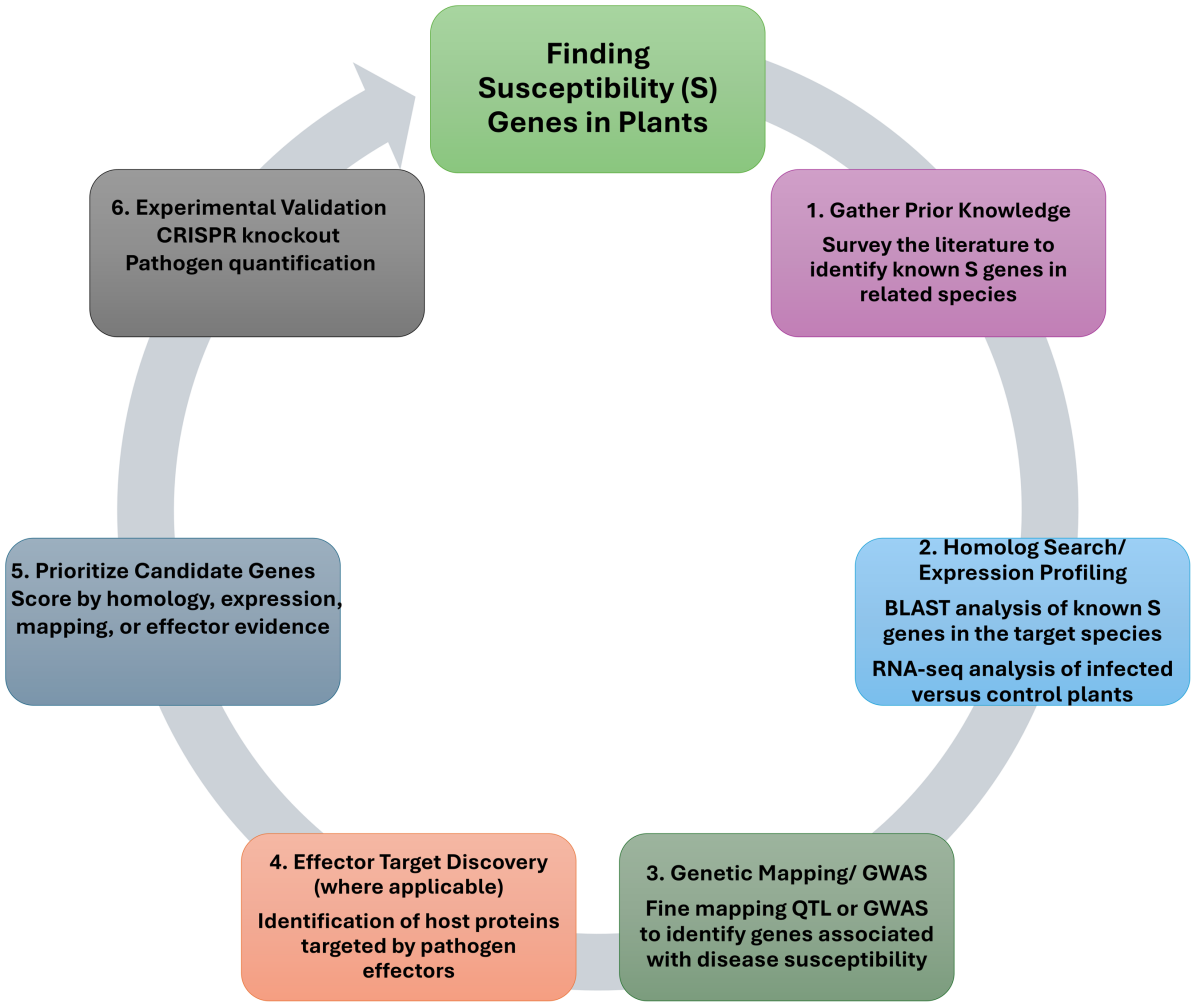
Stepwise framework for identifying susceptibility (S) genes in plants. The initial step involves gathering prior knowledge by reviewing published literature, genomic databases, and previously reported S genes in related species. Such background information provides the basis for analyses in subsequent steps. Identifying homologs and analyzing gene expression involves using BLAST searches to identify orthologs of known S genes and genome-wide expression profiling of infected and healthy plant tissues. These approaches help narrow down genes that may contribute to susceptibility. This step is followed by effector target identification (where applicable) to identify which plant proteins are targeted or manipulated by the pathogen effectors. Since effectors often interact with host susceptibility factors, mapping these interactions provides strong support for selecting S genes. Candidate genes are then prioritized based on sequence conservation, infection-related expression patterns, genetic mapping data, and confirmed effector interactions. The final stage is experimental validation. Using gene knockout or knockdown methods (*e.g.*, CRISPR-Cas), overexpression in transgenic plants, pathogen-inoculation loss-of-function assays, and protein–protein interaction studies, the candidate S genes are tested. Those genes whose editing leads to increased resistance are confirmed as true S genes. This figure outlines an integrated pipeline that combines genomic analysis, transcriptomics, effector biology, and molecular genetics to identify susceptibility genes that can be used to improve plant disease resistance and support crop improvement efforts. Figure created using BioRender.com and Powerpoint.

**Figure 3:**
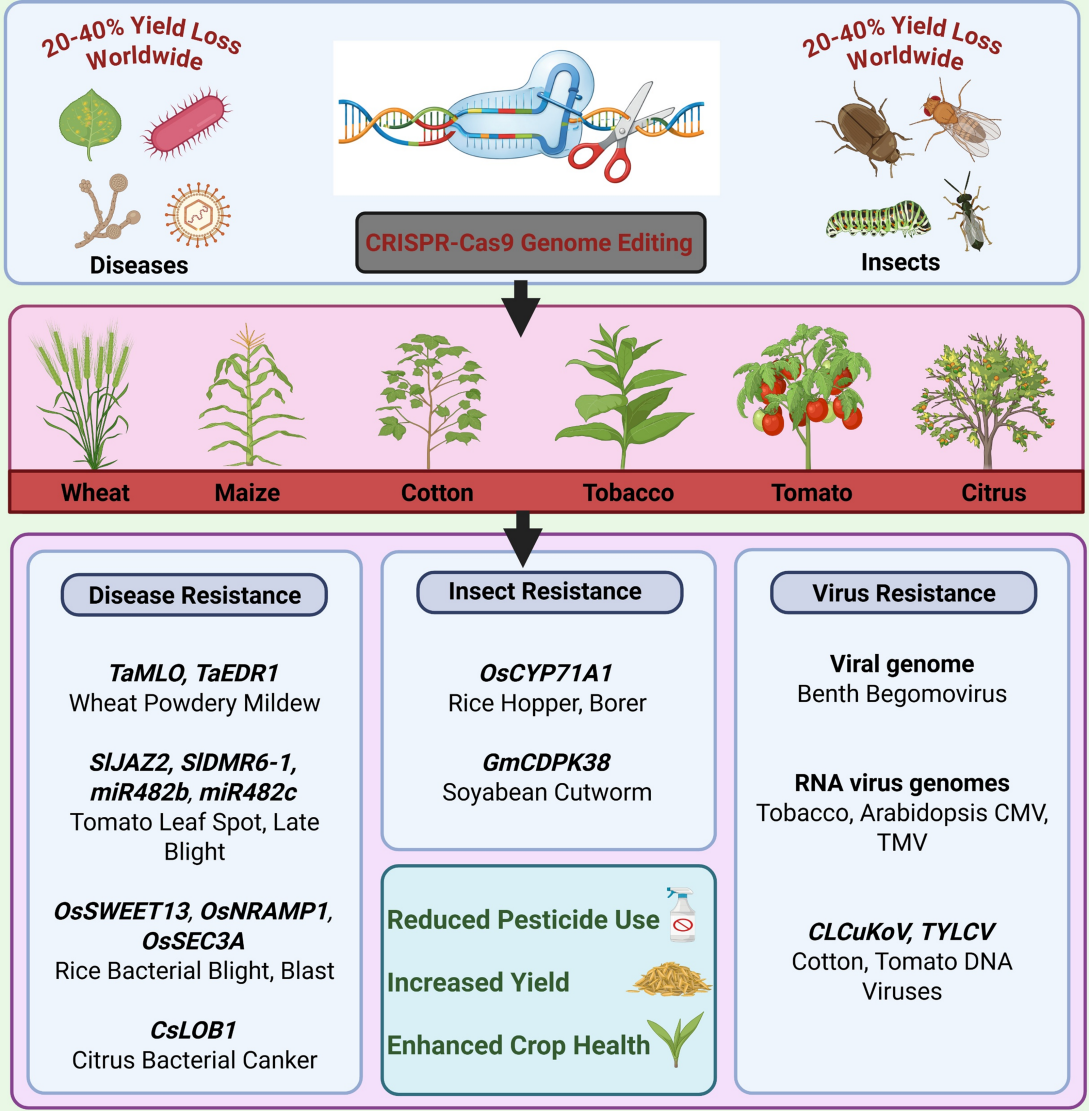
CRISPR-based improvement of biotic stress tolerance in different crops. The technology of CRISPR-Cas9 genome editing is applied to address the loss in crop yields resulting from different types of diseases or pests for important crops including wheat, maize, and tomato *etc*. Identifies key targets that can be edited with CRISPR-Cas9 to create immunity to fungi, bacteria, and viral pathogens. These include *TaMLO* which helps increase the resistance of crops to plant diseases and *OsCYP71A1* which increases the plants immunity to insects. Figure created using BioRender.com and Powerpoint.

In RNA viruses, including *CMV* and *TMV*, the FnCas9-sgRNA complex has been used to directly edit viral RNA genomes. When expressed in tobacco and *Arabidopsis*, it conferred molecular-level resistance to these RNA viruses (Zhang et al., 2018). Moreover, for several DNA viruses such as *CLCuKoV*, *TYLCSV*, and *TYLCV*, CRISPR-Cas9 has been used to interfere with their genome functions (Zaidi et al., 2016). Nevertheless, many susceptibility (S) genes linked to biotic stress have been targeted for editing to enhance tolerance against pests and diseases in various crops (Hussain et al., 2024). Figure 2 presents a roadmap used to identify susceptible (s) genes involved in plant pathogen interactions, outlining how candidate genes are identified, prioritized, and experimentally validated. While Fig. 3 shows applications of CRISPR-Cas9 in engineering disease, insect, and virus resistance in crops.

### CRISPR mediated improvement of abiotic stress tolerance in crop plants

#### CRISPR-Cas mediated improvement of drought tolerance in crops

Drought continues to be the primary abiotic stress restricting crop yield worldwide (Oguz et al., 2022). Classic breeding strategies have achieved limited success in improving drought tolerance, mainly due to its complex, polygenic inheritance. In this regard, genome engineering approaches like the CRISPR-Cas9 system have gained prominence, aimed at revealing gene functions and developing crops with improved drought adaptability (Aleem et al., 2024). Among these, multiplex sgRNA CRISPR-Cas9 systems have been highly effective for genome modification, particularly in monocot species. For example, *TaSal1* mutants were created in wheat cultivar Giza168 using multiplex CRISPR-Cas9 targeting five homologous genes. Several lines exhibited stable, heritable mutations, with five showing complete knockout of all *TaSal1* loci. These mutants demonstrated phenotypes such as stomatal closure, leaf rolling, and improved tolerance to polyethylene glycol-induced stress, showing the robustness of multiplex CRISPR-Cas9 for wheat genome engineering (Abdallah et al., 2025).

*SlGT30*, a tomato trihelix transcription factor, regulates drought response and fruit development. CRISPR disruption of *SlGT30* lowered stomatal density, thereby decreasing water loss and enhancing drought tolerance. Besides, the fruit size and weight were increased by bigger and more numerous pericarp cells in the edited plants; therefore, it presents *SlGT30* as an attractive candidate for breeding purposes (Lv et al., 2025). The *ZmPL1* gene in maize acts as a negative regulator of drought tolerance. The overexpression of *ZmPL1* led to enhanced drought sensitivity, whereas plants knocked out by CRISPR-Cas9 displayed improved germination and survival rates, lower ROS and MDA accumulation, and higher antioxidant enzyme activity under stress. These results indicate that suppression of *ZmPL1* enhances drought resilience in maize (Wang et al., 2025).

Another drought and ABA-inducible gene, *ZmASR1*, has been localized to both the cytoplasm and nucleus. CRISPR-Cas9 knockout of *ZmASR1* led to reduced ROS accumulation, higher ABA levels, and accelerated stomatal closure, all of which contributed to enhanced drought tolerance. This suggests that *ZmASR1* is a negative regulator of drought response and could be a useful target to enhance stress tolerance in maize (Yang et al., 2024). Silencing *OsmiR535*, a microRNA associated with stress signaling, markedly enhanced rice tolerance to dehydration, polyethylene glycol (PEG), ABA, and salinity stress. On the other hand, its overexpression reduced seed survival under dehydration, demonstrating that proper regulation of *OsmiR535* is critical for adaptation to abiotic stress (Yue, Cao & Liu, 2020).

In addition, *ZmHDT103*, which encodes a histone deacetylase, was identified as a drought-responsive gene in maize. The CRISPR-Cas9 knockout and China mutant lines showed lower water loss, ROS, and MDA levels, and higher RWC, ABA concentration, and antioxidant activity under drought stress. Therefore, the results above demonstrate that *ZmHDT103* is a negative regulator of drought tolerance (Wang et al., 2024). Moreover, CRISPR-Cas9 knockout of *ZmGA20ox3*, a crucial player in gibberellin biosynthesis in maize, resulted in semi-dwarf maize mutants exhibiting a significant increase in drought resistance and a relatively small yield penalty compared to the wild type. The levels of ABA, jasmonic acid (JA), and DIMBOA were further elevated in these mutants, confirming *ZmGA20ox3*’s major role in coordinating drought adaptation and growth regulation in maize (Liu et al., 2023).

The first demonstration of CRISPR-Cas genome editing in cotton was carried out by (Li, Unver & Zhang, 2017), who targeted *GhMYB25-like A* and *GhMYB25-like D* genes using the CRISPR-Cas9 system and obtained deletion frequencies ranging from 14.2% to 21.4% at the targeted loci (Li, Unver & Zhang, 2017). Later, Long et al. (2018) designed a novel platform for assessing CRISPR-Cas9 editing efficiency in cotton through the implementation of a transient expression assay. In the system, the endogenous *GhU6* promoter was utilized to drive gRNA expression rather than the *AtU6* promoter. This alteration results in a 6–7-fold increase in sgRNA levels, thereby enhancing mutation efficiency by 4–6 times compared to the AtU6-driven system (Gao et al., 2017). They developed a new platform for evaluating CRISPR-Cas9 editing efficiency in cotton using a transient expression assay. Further, they targeted *GhEF1* and *GhPDS* genes with dual sgRNA expression cassettes, including two sgRNAs within *GhPDS* that induce fragment deletions. Besides, transgenic cotton plants overexpressing *AREB*/*ABF* transcription factors showed enhanced drought tolerance with regulated stomatal conductance and improved photosynthetic efficiency. Transcription factors play key roles in conferring tolerance to drought, salinity, and other abiotic stresses in cotton. For example, overexpression of *GhABF2*, a bZIP transcription factor, significantly enhanced drought and salt tolerance by regulating ABA-responsive signaling pathways. Moreover, *GhABF2* overexpressed lines showed better yield performance as compared to non-transgenic cotton plants (Ullah et al., 2018).

Likewise, the heterologous expression of *DgCspC* was reported to enhance photosynthetic efficiency and yield in cotton. The *DgCspC* gene increased the accumulation of osmolytes and stress-related metabolites, thus facilitating osmotic adjustment and ROS scavenging under stressful conditions (Xia et al., 2022). The *GHSP26* gene from *Gossypium arboreum*, which encodes a small heat-shock protein, was transformed into *G. hirsutum* to enhance drought tolerance. The transgenic lines showed greater drought resistance than the wild type, indicating that *GHSP26* plays a functional role in stress adaptation (Maqbool et al., 2010). Applications of CRISPR-mediated genome editing to enhance abiotic stress tolerance in crops toward climate resilience are summarized in Table 3, which presents CRISPR-based improvements for salt, drought, heat, and cold stresses.

**Table 3:** Applications of CRISPR-mediated genome editing for enhancing abiotic stress tolerance in crops toward climate resilience.

**Crop**	**Target gene(s)**	**Editing approach**	**Stress type**	**Key outcome**	**Reference**
Wheat	*TaSal1*	CRISPR-Cas9 knockout (multiplex)	Drought	Mutants showed closed stomata and enhanced tolerance to PEG stress	Abdallah et al. (2025)
Tomato	*SlGT30*	CRISPR-Cas9 knockout	Drought	Reduced stomatal density and improved drought tolerance	Lv et al. (2025)
Maize	*ZmPL1*	CRISPR-Cas9 knockout	Drought	Reduced ROS, increased germination and survival rates	Wang et al. (2025)
Maize	*ZmASR1*	CRISPR-Cas9 knockout	Drought	Enhanced ABA levels and stomatal closure under stress	Yang et al. (2024)
Rice	*OsmiR535*	CRISPR-Cas9 suppression	Drought & Salt	Improved tolerance to dehydration, PEG, ABA, and salt stress	Yue, Cao & Liu (2020)
Maize	*ZmHDT103*	CRISPR-Cas9 knockout	Drought	Reduced water loss and ROS, improved ABA and antioxidant activity	Wang et al. (2024)
Maize	*ZmGA20ox3*	CRISPR-Cas9 knockout	Drought	Semi-dwarf mutants showed enhanced drought tolerance	Liu et al. (2023)
Rice	*OsDST*	CRISPR-Cas9 knockout	Drought & Salt	Mutants showed reduced stomatal density and improved tolerance	Kumar et al. (2020)
Soybean	*GmARM*	CRISPR-Cas9 knockout	Salinity & Pathogen	Enhanced tolerance to saline-alkali stress and root rot	Luo et al. (2024)
Rice	*OsDSG1*	CRISPR-Cas9 knockout	Salt	Improved salt tolerance and reduced oxidative stress	Ly et al. (2024)
Barley	*ITPK1*	CRISPR-Cas9 knockout	Salt	Enhanced salt tolerance phenotype	Vlčko & Ohnoutková (2020)
Rice	*OsRbohB*	CRISPR-Cas9 knockout	Heat	Improved chlorophyll, yield, and stress gene expression under heat stress	Liu et al. (2025)
Rice	*OsNAC050*	CRISPR-Cas9 knockout	Cold	Enhanced cold tolerance through stress pathway modulation	Yarra & Wei (2021)
Rice	*OsAnn5*	CRISPR-Cas9 knockout	Cold	Increased seedling survival under cold stress	Que et al. (2023)
Maize	*ZmPL1*	CRISPR-Cas9 knockout	Drought	Reduced ROS, increased germination and survival rates	Wang et al. (2025)

#### CRISPR-Cas mediated improvement of salt tolerance in crops

Across the world, soil salinity represents a critical challenge to sustaining crop production. It affects more than a billion hectares in more than 100 countries and is still on the rise (Atta et al., 2023). Due to the persistence of soil salinity, increasing plant tolerance against salt stress has become an important objective in crop improvement programs. The recent development of genome editing, especially CRISPR-Cas mediated systems, now allows for precise modification of genes involved in stress adaptation and opens new avenues for developing crops that are resilient to salinity. A wide array of salt-inducible genes and transcription factors has been identified, and their genetic manipulation has been used to enhance salinity stress tolerance. These genes have been involved in ion homeostasis, osmotic balancing, hormone signaling, and reactive oxygen species processing-all critical processes that allow plants to survive under saline conditions. *OsDST* is a rice zinc-finger transcription factor that plays a significant role in controlling stomatal behavior under drought and salinity stress. In a recent study, CRISPR-Cas9 mediated *OsDST* knockout in indica rice MTU1010 resulted in fewer but larger stomata. This reduced transpiration loss and ultimately enhanced the plants’ tolerance to both drought and salt stress (Kumar et al., 2020). Mutant named Pusa DST rice1 became one of India’s earliest officially released genome-edited rice varieties. It showed a 10–30% yield advantage under stress conditions while maintaining its original grain quality (Kumar et al., 2020).

*GmARM* acts as a negative regulator in soybeans under saline and alkaline stresses, as well as in Phytophthora root rot. It controls the plant stress responses *via* both ABA-responsive elements and MYB-binding site pathways, integrating relevant signaling networks of abiotic and biotic stresses. The soybean plants with CRISPR-Cas9 mediated knockout of *GmARM* showed enhanced tolerance to salinity, alkalinity, and pathogen attack, with better survival rates and physiological stability than wild-type plants, confirming its role in increasing stress susceptibility (Luo et al., 2024).

Similarly, targeted mutations in the *OsDSG1* gene, which controls ubiquitination and stress-responsive signaling in rice, conferred enhanced salt tolerance and improved growth performance, along with reduced oxidative damage (Ly et al., 2024). The *DST* gene was mutated in the indica cultivar MTU1010 using two guide RNAs, targeting its protein–protein interaction domain. This editing event resulted in a 366 bp deletion and gave rise to plants with wider leaves and reduced stomatal density. Loss of function lines depicted moderate tolerance to osmotic stress and strong tolerance to high salinity, confirming that *DST* functions negatively influence the plant’s ability to tolerate salinity (Ly et al., 2024).

Further research showed that CRISPR-Cas9 has the potential to enhance salt tolerance by specifically editing genes encoding phosphatases and kinases, such as *ITPK1* in barley (Vlčko & Ohnoutková, 2020), *SAUR41* in *Arabidopsis*, and *HAG1* in wheat. Suppression of *OsRR22*, a central cytokinin signaling regulator, significantly improved salt stress tolerance in rice (Choudry et al., 2024).

However, these examples demonstrate the transformative role of CRISPR-Cas based genome editing in studying and enhancing plant responses to salt stress. Precise targeting of genes that govern ion transport, signaling, transcriptional regulation, and redox balance enables genome editing to expedite the development of salt and drought tolerant varieties for sustainable agricultural productivity under climate-stress conditions.

#### CRISPR-Cas mediated regulation of temperature stress responses

Increased temperatures, along with fluctuating climatic conditions, result in significant reductions in crop productivity, particularly in temperature-sensitive crops such as rice, tomato, and soybean. Heat stress disrupts major physiological and molecular processes related to oxidative damage and impaired photosynthesis, contributing to yield reduction (Park, Kim & Kim, 2025). The genome-editing platforms, particularly CRISPR-Cas systems, have become valuable tools for identifying and modifying temperature-responsive genes, enabling researchers to determine whether these genes enhance or suppress thermotolerance (Kumar et al., 2023).

##### Heat stress responses.

Thus, calcium signaling initiates downstream antioxidant defenses in response to heat stress. In tomato, disruption of *CPK28* using CRISPR-Cas9 revealed that *cpk28* mutants were more sensitive to high temperatures, with higher ROS levels and reduced APX activity. Treatment of these mutants with reduced ascorbate partly reestablished their heat tolerance, suggesting that *CPK28* promotes thermotolerance by targeting the phosphorylation of APX and strengthening the antioxidant protection system of the plant (Hu et al., 2021). Transgenic CRISPR-Cas9 knockout lines of *OsNAC006* showed increased sensitivity to high temperature, demonstrating that this gene functions as a positive regulator of heat tolerance (Wang et al., 2020b; Wang et al., 2020a). Rice mutants with edits in *OsGER4* produced significantly fewer crown and lateral roots under heat stress, demonstrating that *OsGER4* acts as a negative regulator (Nguyen et al., 2023). Moreover, HSPs are among the major contributors to thermotolerance, helping stabilize and refold denatured proteins under heat stress (Razzaq et al., 2023).

**Figure 4:**
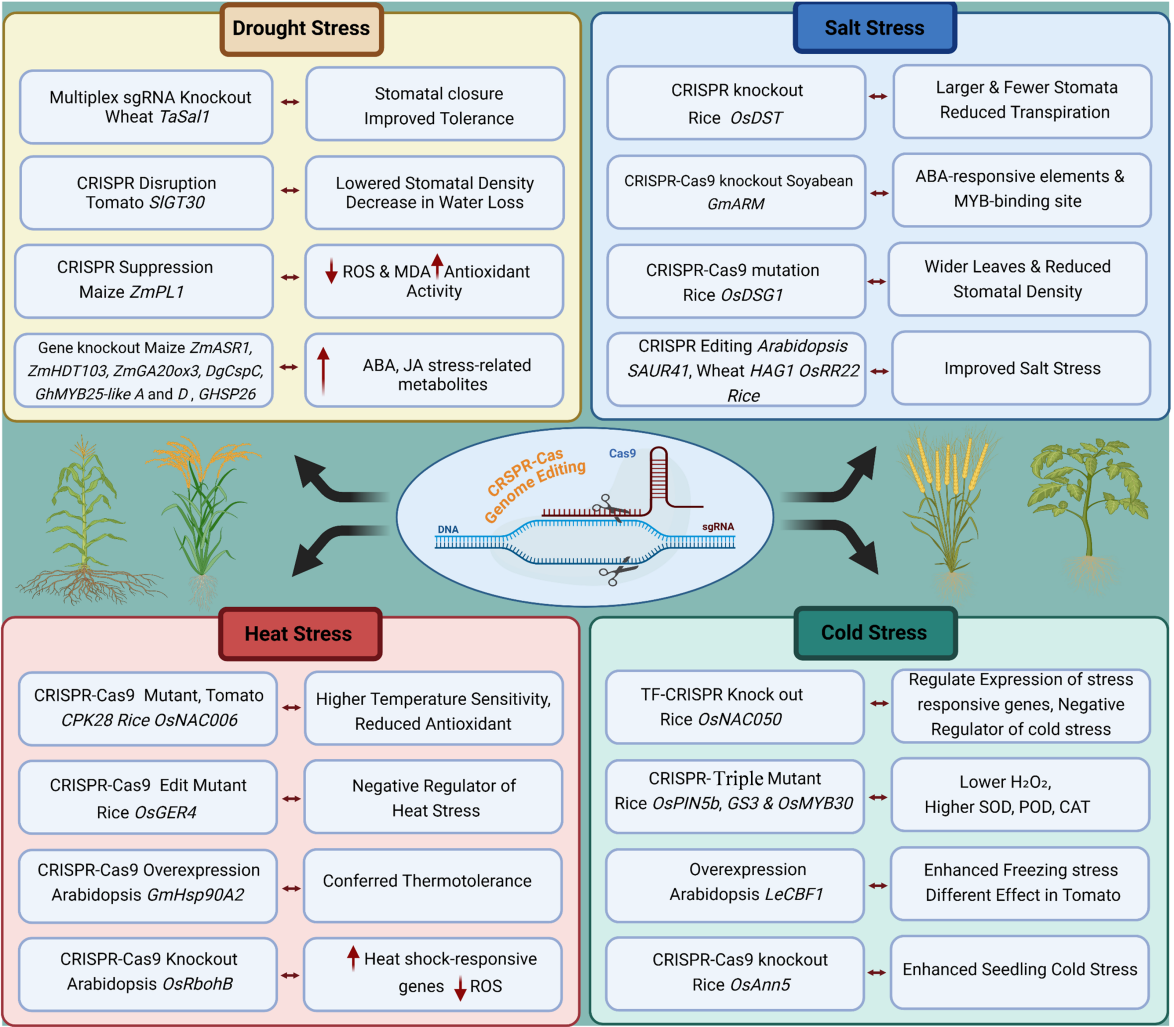
CRISPR-based improvement of abiotic stress tolerance in different crops. The CRISPR-Cas9 tool has been strategically applied to increase the tolerance of abiotic stresses in key crops through targeted genetic pathways responsible for tolerance to drought, salinity, high temperatures, and low temperatures. This is achieved through multiple gene editing approaches, including the knockout of genes like *TaSal1* and *OsDST*, which help control stomata movement; as well as the upregulation of genes like *GmHsp90A2* to increase thermotolerance. Figure created using BioRender.com and Powerpoint.

Twelve *GmHsp90* genes were identified in soybean, one of which, *GmHsp90A2*, was overexpressed in Arabidopsis and conferred remarkable thermotolerance (Xu et al., 2013). In rice, CRISPR-Cas9 mediated knockout of *OsRbohB*, a key NADPH oxidase gene, conferred heat tolerance at multiple developmental stages. The *OsRbohB*-KO lines have lower accumulation of ROS, higher chlorophyll content, increased yield, and upregulation of heat shock-responsive genes, thus providing a new approach to reduce yield loss due to heat (Liu et al., 2025). Figure 4 shows targeted genome editing in various crops against abiotic stresses.

##### Cold stress responses.

*OsNAC050*, one of the NAC transcription factors of rice, participates in regulating cold tolerance. CRISPR-based knockout of *OsNAC050* substantially improved seedling cold tolerance by modulating the expression of stress-responsive genes. These findings indicate that *OsNAC050* acts as a negative regulator of cold tolerance during the seedling stage and could serve as a promising target for developing cold-resistant rice varieties through CRISPR genome editing (Yarra & Wei, 2021; Zeng et al., 2020) applied CRISPR-based multiplex genome editing to simultaneously target *OsPIN5b*, *GS3*, and *OsMYB30*, which regulate panicle length, grain size, and cold tolerance, respectively. The resulting triple mutants, ospin5b/gs3/osmyb30-4 and ospin5b/gs3/osmyb30-25, exhibited enhanced agronomic traits, along with increased yield and improved cold resilience. Their survival rates were 70.8% and 79.1%, respectively, when evaluated at low temperature (4 °C), as compared to 45.8% for the wild type (Zeng et al., 2020). These mutants showed lower accumulation of superoxide and hydrogen peroxide (H_2_O_2_) and higher activities of antioxidant enzymes, such as SOD, POD, and CAT. This enhanced ROS-scavenging ability contributed to improved detoxification and enhanced survival under cold-stress conditions (Wang et al., 2023).

In tomato, three CBF homologs, *LeCBF1-3,* have been identified, of which only *LeCBF1* is cold-inducible and thus restricts cold acclimation. Overexpression of *LeCBF1* enhanced freezing tolerance in Arabidopsis. Still, it did not cause a similar increase in tomato, indicating that the CBF regulon in tomato has less diversity than in Arabidopsis and thus acquired only limited cold tolerance (Zhang et al., 2004). Furthermore, H_2_O_2_ acts as a signaling molecule to enhance chilling tolerance in tomato by activating stress-responsive pathways. Pretreatment with one mM H_2_O_2_ upregulated the expression of *SlCBF1* and *SlMAPK1/2/3,* modulated hormone levels, including increased methyl jasmonate (MeJA), zeatin riboside (ZR), and abscisic acid (ABA), but decreased the concentration of gibberellic acid (GA_3_), and collectively enhanced the resilience against cold stress (Wang et al., 2017). Light and hormonal signals regulate *OsAnn5* in rice, which contains unique promoter elements, including DRE and MYB recognition sites. CRISPR-Cas9 knockout of *OsAnn5* markedly enhanced seedling survival under cold stress, suggesting that it positively regulates cold tolerance (Que et al., 2023). Hence, these studies demonstrated that CRISPR-based genome engineering has the potential to elucidate plant responses to temperature extremes by targeting key regulators, including antioxidant-related genes, transcription factors, and kinases.

### Challenges and future perspectives of CRISPR in sustainable agriculture

Despite the important progress in CRISPR-based development of biotic and abiotic stress tolerance, several regulatory, technical, and socio-economic constraints remain that must be addressed to fully harness its potential in sustainable crop production. Table 4 highlights the major constraints and forward-looking opportunities that will shape the future application of CRISPR in sustainable crop improvement. Coordination of biosafety guidelines, participation in public communication, and fair access to genome editing technologies would provide the necessary framework for reasonable acceptance of these technologies.

**Table 4:** Challenges and future perspectives of CRISPR in sustainable agriculture.

**Category**	**Major challenge**	**Possible solutions**	**Future outlook**	**References**
Technical	Off-target mutations	Use of high-fidelity Cas variants	Greater precision and safety	Abdallah et al. (2025)
Delivery Methods	Limited transformation efficiency	Nanoparticle, viral vector, and RNP delivery	Wider applicability in recalcitrant crops	Chen et al. (2024)
Regulatory Barriers	Unclear legal status in many countries	Science-based, harmonized regulation	CRISPR-edited crops treated as non-GMO	Chen et al. (2024)
Ethical & Public Acceptance	Perception of “genetic modification.”	Transparent communication, risk assessment	Wider adoption through education	Chen et al. (2024); De Guimaraes, Tsang & Cabral (2025)
Integration with Breeding	Limited breeder training	Cross-disciplinary programs	CRISPR-integrated modern breeding systems	Abdallah et al. (2025)
Sustainability	Dependence on a few crops	Editing for orphan crops	Diversified, climate-resilient agri-ecosystems	Khanna, Ohri & Bhardwaj (2023); Ahmar et al. (2024)

## Conclusion

CRISPR-mediated genome editing has enabled precision and efficiency in modifying genes by controlling stress adaptation, yield, and nutritional traits for crop improvement. It therefore enables researchers to decipher the intricate genetic networks that control plant responses to stress, such as drought, salinity, heat, and pathogen attack, with a view to engineering cultivars that can thrive under extreme environmental conditions. CRISPR accelerates the development of climate-resilient crops much faster and more accurately than any conventional breeding approach by precisely modulating critical stress-responsive genes such as *DREB, HSP, NAC, SOS*, and *NHX*. This technology, combined with multi-omics platforms, high-throughput phenotyping, and AI-driven data analytics, holds immense potential to reshape the future of sustainable agriculture and strengthen global food security. Full-scale application of CRISPR in agriculture will, in the future, depend on how efficiently several challenges, including off-target activity, improved delivery efficiency, and consideration of ethical and regulatory frameworks, are addressed. Table 4 highlights the major constraints and forward-looking opportunities that will shape the future application of CRISPR in sustainable crop improvement. Coordination of biosafety guidelines, participation in public communication, and fair access to genome editing technologies would provide the necessary framework for reasonable acceptance of these technologies. Successive research efforts should be directed toward multiplex editing, base and prime editing, and CRISPR mediated regulation to provide fine-tuning of complex traits without introducing foreign DNA. Collaboration among molecular geneticists, plant breeders, policymakers, and data scientists will be very important for using into the full potential of CRISPR. Collectively, all these will prepare the ground for a globally resilient, productive, and ecologically friendly food production system that fulfills the United Nations’ 2030 Sustainable Development Goals.

##  Supplemental Information

10.7717/peerj.21450/supp-1 Supplemental Information 1 CRISPR-mediated precision genome editing for developing climate-resilient crops and enhancing food security Figure created using BioRender.com and Powerpoint

## Additional Information and Declarations

### Data Availability

The following information was supplied regarding data availability: This is a literature review.
