# Supplementary figures and images for: Clustered Regularly Interspaced Short Palindromic Repeats (CRISPR)-Cas genome editing transforming crop stress tolerance for global food security
Source: PeerJ. 2026 Jul 1;14:e21450. doi: 10.7717/peerj.21450 (PMC13332720; doi:10.7717/peerj.21450)

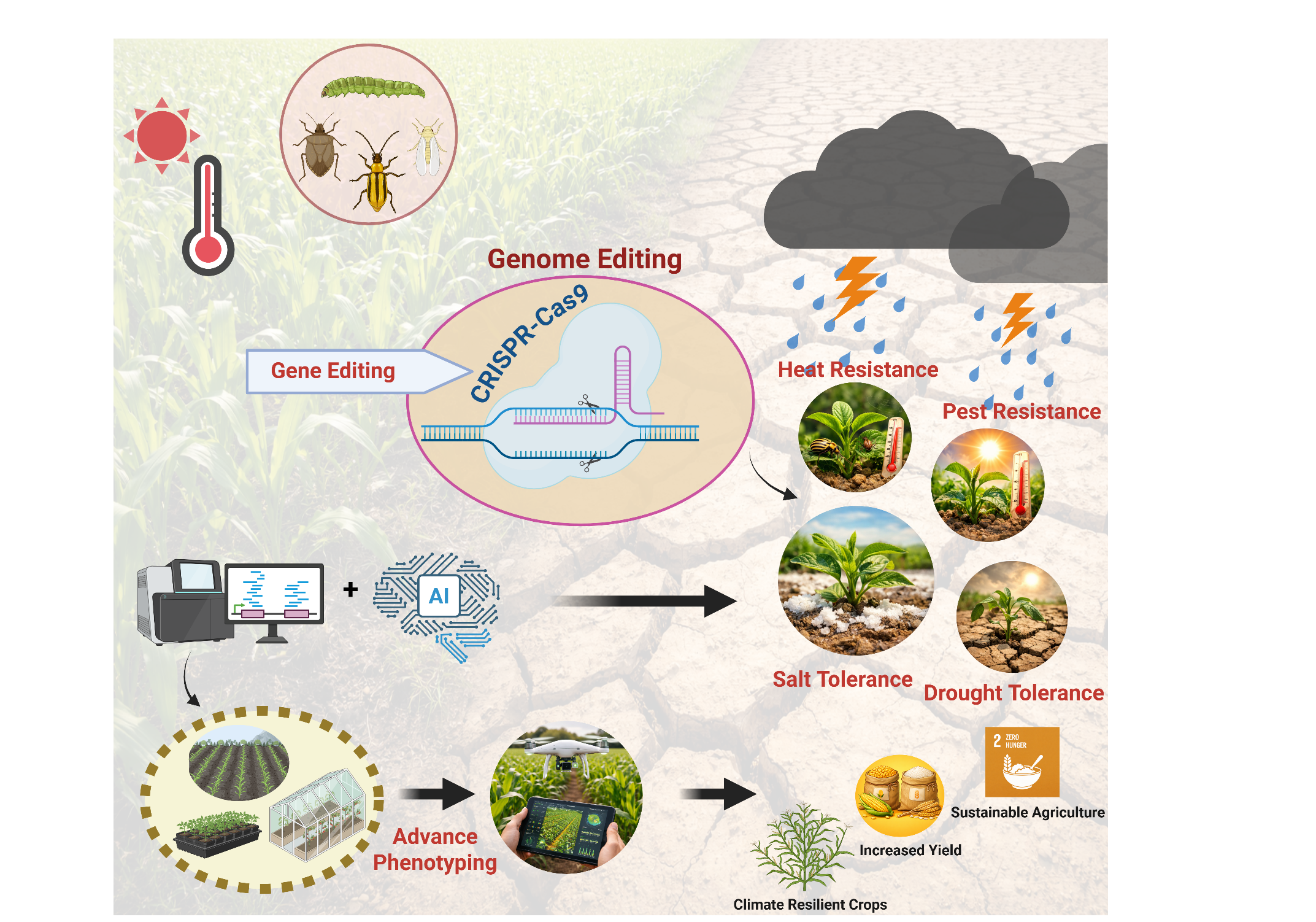

Supplement: Supplemental Information 1 — Figure created using BioRender.com and Powerpoint [file peerj-14-21450-s001.png]
